# microRNAs Associated with Carotid Plaque Development and Vulnerability: The Clinician’s Perspective

**DOI:** 10.3390/ijms232415645

**Published:** 2022-12-09

**Authors:** Rafał Badacz, Tadeusz Przewłocki, Jacek Legutko, Krzysztof Żmudka, Anna Kabłak-Ziembicka

**Affiliations:** 1 Department of Interventional Cardiology, Institute of Cardiology, Jagiellonian University Medical College, św. Anny 12, 31-007 Kraków, Poland; 2 Department of Interventional Cardiology, The John Paul II Hospital, Prądnicka 80, 31-202 Kraków, Poland; 3 Department of Cardiac and Vascular Diseases, Institute of Cardiology, Jagiellonian University Medical College, św. Anny 12, 31-007 Kraków, Poland; 4 Noninvasive Cardiovascular Laboratory, The John Paul II Hospital, Prądnicka 80, 31-202 Kraków, Poland

**Keywords:** atherosclerosis, atherosclerotic risk factors, carotid artery stenosis, carotid plaque, cerebrovascular ischemia, endothelial cells, microRNAs, plaque vulnerability, platelets, vascular smooth muscle cells

## Abstract

Ischemic stroke (IS) related to atherosclerosis of large arteries is one of the leading causes of mortality and disability in developed countries. Atherosclerotic internal carotid artery stenosis (ICAS) contributes to 20% of all cerebral ischemia cases. Nowadays, atherosclerosis prevention and treatment measures aim at controlling the atherosclerosis risk factors, or at the interventional (surgical or endovascular) management of mature occlusive lesions. There is a definite lack of the established circulating biomarkers which, once modulated, could prevent development of atherosclerosis, and consequently prevent the carotid-artery-related IS. Recent studies emphasize that microRNA (miRNA) are the emerging particles that could potentially play a pivotal role in this approach. There are some research studies on the association between the expression of small non-coding microRNAs with a carotid plaque development and vulnerability. However, the data remain inconsistent. In addition, all major studies on carotid atherosclerotic plaque were conducted on cell culture or animal models; very few were conducted on humans, whereas the accumulating evidence demonstrates that it cannot be automatically extrapolated to processes in humans. Therefore, this paper aims to review the current knowledge on how miRNA participate in the process of carotid plaque formation and rupture, as well as stroke occurrence. We discuss potential target miRNA that could be used as a prognostic or therapeutic tool.

## 1. Introduction

Ischemic stroke (IS) is one of the leading causes of mortality and disability in developed countries [1]. Atherosclerotic internal carotid artery stenosis (ICAS) accounts for about 20% cases of cerebral ischemia [2]. The present diagnostic tools for carotid artery assessment are based on imaging studies, including carotid Doppler ultrasonography, computed tomography, magnetic resonance, or conventional invasive angiography with a use of intravascular ultrasound (IVUS), and optical coherence tomography (OCT) [3,4,5]. They display the degree of ICAS, as well as carotid plaque morphology [6].

The current guidelines position carotid endarterectomy (CEA) and carotid artery stenting (CS) as the established treatment methods for ICAS [7]. In addition to invasive treatment, the optimal medical approach, including cardiovascular risk factor-control, as well as pharmacotherapy (i.e., antiplatelet and antidiabetic agents, lipid and blood pressure lowering medication), should be introduced in order to reduce IS risk [8,9]. The optimal timing for the intervention on carotid artery is controversial [10,11]. According to guidelines, CEA or CS is recommended in recently symptomatic ICAS with stenosis severity above 50% lumen reduction [10,11], whereas the intervention on asymptomatic ICAS is recommended in high-grade stenosis, or in carotid plaques exceeding 60% lumen reduction when features of high-risk plaque for cerebral ischemia are present [10]. As IS can result from a fragmented plaque debris release with a subsequent embolization of cerebral arteries, plaque rupture followed by local carotid artery thrombosis, or hypoperfusion of cerebral structures, the mechanism of cerebral ischemia is complex [12,13,14,15]. Thus, as evidenced, plaque morphology and structure, in addition to the degree of carotid stenosis, play the pivotal role in the IS risk assessment and decision on the intervention [16].

The serious drawback of the aforementioned imaging tools is that they do not allow for the assessment of early stages of atherosclerosis, i.e., those that precede intima-media complex thickening and early fatty lesions incidences [17]. Unfortunately, current guidelines miss laboratory biomarkers which could predict the incidence of IS and thus target the high-risk group of patients with preemptive treatment, whereas early intervention upon the initiation of atherosclerosis seems very attractive [18]. Data show the important roles of pro-atherothrombotic and pro-inflammatory biomarkers, including cytokines (IL-1β, IL-6, TNFα), platelets, and macrophages activity [19,20,21].

Recent studies emphasize that microRNA (miRNA) are the emerging particles that could potentially play a pivotal role in this approach [22]. miRNAs are small, non-coding RNA nucleotides, having a length that is typically between 18 and 27 nucleotides that regulate post-transcriptional gene expression, by binding to the 3′- (more often), or to 5′-untranslated regions of mRNA, or exons [23]. The role of the miRNA has already been confirmed in the broad range of both physiological and pathological processes [24]. They are responsible for target gene expression regulation after the transcription process, either by inhibiting the translation or mRNA degradation [25]. The diagnostic and prognostic role of circulating miRNAs in ICAS leading to IS has been studied, however the conclusions remain inconsistent. This paper aims to review the current knowledge and assess the newest studies on how the miRNA participate in the process of carotid plaque formation and rupture, as well as stroke occurrence.

## 2. From Fatty Streaks and Foam Cells to Mature Plaque

Plaque formation initiates from stages that are not detectable by imaging tools [26]. First stages include endothelium dysfunction, accompanied by inflammation and modified low-density lipoprotein (LDL) retention in the intimal layer of the intima-media complex [27]. In the endothelium equilibrium, a great number of miRNAs are involved, including protective ones [28]. Their protective effect is achieved through many signaling pathways, however their major role is to prevent unfavorable lipid metabolism and reduce inflammation [28]. One of these miRNAs, miR-126, protects endothelial cells (ECs) through the suppression of NOTCH-1 inhibitor and activation of the vascular endothelial growth factor (VEGF) signaling (Table 1) [29,30]. At the beginning, miR-155 induces the downregulation of mitogen-activated protein 3 kinase 10 (MAP3K10), endothelin-1 (ET-1), and angiotensin II (ANG II) type I receptor [31,32]. The downregulation of ET-1 is important in many cardiovascular settings, as elevated levels of ET-1 are independently associated with increased cardiovascular mortality [33,34]. miR-146a and miR-125a decrease the lipid uptake in macrophages [35,36]. miR-146a also inhibits endothelial activation by increasing nitric oxide synthase (eNOS) expression [35]. miR-125 modulates extracellular vascular endothelial growth factor (VEGF) by manipulating macrophage soluble VEGF receptor-1 (sVEGFR1) production. This mechanism has a therapeutic potential in many diseases [36]. miR-206 and miR-223 regulate cholesterol synthesis through the reverse cholesterol transport from macrophages to the liver for excretion, attenuates pro-inflammatory cytokine production, and has a role in platelet activation [37,38,39,40,41].

Some miRs, such as miR-let-7g, also modulate ECs senescence by regulating anti-aging gene sirtuin 1 (SIRT1) and the insulin growth factor (IGF) 1 pathway [42]. In line with this, miR-143 is downregulated with advancing age and protects against vascular senescence [43,44]. On the contrary, miR-92a released from ECs stimulates macrophages to the pro-inflammatory responses and LDL uptake, which enhance atherosclerotic progression [85]. In mice, inhibition of miR-92a reduces endothelial inflammation and atheroma plaque size through the regulation of Kruppel-like factor 2 (KLF2) [47]. Similarly, miR-34a aggravates and accelerates vascular senescence through the downregulation of SIRT1 and AXL Receptor Tyrosine Kinase [48], whereas miR-34a inhibition by anti-miR-34a reduced vascular inflammation, senescence, and apoptosis [49,86]. In macrophages, ox-LDL increases miR-34a levels that target the cholesterol transporters’ ATP-binding cassette subfamily A member 1 (ABCA1), and ATP-binding cassette subfamily G member 1 (ABCG1) [48]. This alters lipid metabolism, while miR-34a enhanced secretion of inflammatory cytokines promotes inflammation facilitating atherosclerotic plaque formation [48,49]. ECs dysregulation is enhanced by the lipid accumulation due to disturbed reverse cholesterol transport of cholesterol efflux from macrophages to the liver [50]. This results in lipid accumulation in macrophages with formation of foam cells [48]. miR-206 and miR-233 promote cholesterol efflux to the liver, whereas miR-33a inhibits reverse transport (Table 1) [39,40,51,87]. miR-33a/b have been shown to act as post-transcriptional regulators of a lipid metabolism, and their pharmacological inhibition diminished atherosclerosis by raising plasma high-density lipoprotein levels [87]. Nguyen et al. demonstrated that chitosan nanoparticles containing miRs can be delivered to macrophages [88]. In mice, macrophages treated with miR-33-loaded nanoparticles showed decreased reverse cholesterol transport [88]. In contrast, when efflux-promoting miRs were delivered the efflux was improved [88].

miR-10a, miR-31, and miR-17-3p regulate inflammation modulating the expression of adhesion molecules in ECs, while miR-155 and miR-331 through the down-regulation of the anti-inflammatory suppressor of cytokine signaling 1 (SOCS1) protein [53,54,73,89,90]. During atherosclerosis development, miR-155 begins to stimulate atherosclerosis progression through repressing Bcl6 in macrophages, suppressing the expression of eNOS, and increasing pro-inflammatory NF-κB signaling [52].

There is a continuous crosstalk between ECs and vascular smooth muscle cells (VSMCs) [91]. ECs-derived miRNAs, like miR-126, miR-92a exert action on VSMCs, resulting in the VSMCs-enriched miRNAs release, that often have reciprocal unfavorable effects on ECs [92,93]. On atherosclerosis initiation, VSCMs migrates from the medial arterial wall into the intimal space, resulting in promotion of plaque formation [91]. VSCMs migration and proliferation is one of key stages in early atherosclerosis. miR-520 regulates VSMCs function by targeting RelA/p65. This way, decreasing cells migration and proliferation exerts a protective role in atherosclerosis [45]. Moreover, miR-520c-3p mimics may act as a promising therapeutic strategy for atherosclerosis [45]. The VSMCs equilibrium plays a great role in the inhibition of atherosclerosis. The maintenance of contractile phenotype prevents atherosclerosis [45,59,60,91,92,93,94]. Of the miRs capable of the contractile function recovery in VSMCs, miR-22 and miR143/145 are probably the most investigated ones, and are potential therapeutic targets [59,60,91,94]. Intravenous delivery of miR-143/145 extracellular vesicles blocked atherosclerotic lesion progression and exerted protective effects on intima-media complex [59,60], while miR-22 restores contractile phenotype of VSMCs without a negative impact on EC’s function [95]. In addition, miR-22 inhibits intima-media hyperplasia, which is important both for inhibition of atherosclerosis plaque growth, as well as in the restenosis following stent implantation [46,95].

Some miRNAs were investigated in the context of carotid plaques, including miR-520, miR-455, and miR-105. Some are common for many arterial territories, including miR-21, miR-27, miR-100, and miR-122 (Table 1) [45,55,56,57,58,61,62,63,64,65,66,67,68,69,70,71,72,74,75,76,77,78,79,80,81,82,83,84,96,97,98,99,100,101,102].

When the anti-atherothrombotic miRNAs are overbalanced by the pro-atherothrombotic miRNAs, we can steadily observe carotid intima-media complex thickening (CIMT), followed by the occurrence of focal non-calcified lesions [102]. Then, a formation of mature plaques composed of lipid and necrotic cores, fibrotic matrix, and calcifications are observed, accompanied by inflammation and angiogenesis. The first caution that should attract the attention of clinicians is CIMT [103,104,105]. CIMT, in ranges above the 75th percentile for age and gender, can even be observed in teenagers and young adults, particularly if accompanied by atherosclerosis risk factors, such as diabetes or familial hypercholesterolemia [106,107]. This parameter is well correlated with risk of cardiovascular events, such as cardiovascular death (CVD), IS, and myocardial infarction (MI) [103,104,105]. It has also a good predictive value for a presence of significant atherosclerosis in the other territories, e.g., coronary arteries [108,109]. Several miRNAs are associated with CIMT, including miR-22, miR-29a, miR-143/145, and miR-92a [46,70,110,111]. With increasing CIMT, atherosclerotic process accelerates. There is a huge role for metalloproteinases (MMP), such as MMP-2 and MMP-9, as they are associated with a promotion of plaque growth and CIMT increase, rather than a decrease in VSMCs contractility [111]. Interestingly, in advanced carotid plaques, migration and proliferation of VSMCs is beneficial, promoting the stability of the fibrous cap and prevention of plaque rupture [91,102]. This process in stimulated by the expression of miR-145 and miR-210 that drive the increase in plaque collagen content and a fibrous cap area, while at the same time reducing the necrotic core area [46,65,95].

In contrast, plaque instability is associated with increasing levels of MMP-2, MMP-9, the increasing size of plaque and the lipid and necrotic core, particularly when abundant in lipids [111,112,113,114]. MMP-9 is particularly important as it predicts future adverse cardiovascular events [71,111,112,113]. It was observed that MMP-9 is regulated by several miRNAs, including miR-92a, which is a predictor of plaque instability [114]. However, miR-92a is not necessarily always negative [115,116]. The upregulation of MMP-9 and the downregulation of TIMP3 in H_2_O_2_-induced VSMCs were observed to be reversed by mimicking miR-92a in addition to SIRT1 and siRNA, which may prevent a phenotypic change of VSMCs [115,116]. Other miRNAs associated with serum concentration of MMP-9 include miR-100, miR-155, miR-133a, and miR-223 [111,114]. These miRNAs are also linked to plaque instability and might be used as biomarkers of plaque conversion from a stable state into a vulnerable state [114,115,116,117,118,119].

Thus, it is of the utmost importance to identify carotid plaques that are likely to undergo transformation from the stable ones to vulnerable ones, prone to rupture and cause symptoms of cerebral ischemia. The research is ongoing for the identification of specific microRNAs that could prevent IS through the manipulation of their expression levels.

## 3. Degree of Carotid Plaque, Patients with Asymptomatic and Symptomatic ICAS: Prognostic miRNAs

One of the key issues addressed in recent studies was the role of miRNAs in differentiating patients with stable asymptomatic ICAS from those who may develop symptomatic ICAS, thus the identification of patients characterized with high risk of ICAS-related IS [114,119]. Alas, the majority of studies on carotid atherosclerosis describe carotid lesions that are non-occlusive, whereas the occlusive carotid artery lesions regarding miRNA expression profile and levels are under-investigated. Notably, ICAS causes at least a 50% stenosis, which increases the risk of carotid lesion-related IS [120]. miRNA as potential markers of carotid plaque that is non-obstructive compared to plaques causing a significant carotid stenosis (equal or greater than 50% lumen reduction) were investigated by Raskurazhew et al. (Table 2) [100]. In this study, the levels of miR-126-3p, miR-126-5p, miR-21-3p, and miR-29a-3p were higher in non-obstructive carotid plaques, meanwhile they were low in carotid plaques which caused lumen reduction of 50% or greater [100]. In contrast, levels of miR-33a were lower in early carotid plaques, while higher in asymptomatic ICAS exceeding 50% [100].

In a study by Zhu et al., low serum levels of miR-455 were highly diagnostic for a high-grade asymptomatic ICAS, with the AUC value of 0.992, a sensitivity of 82.9%, and a specificity of 90.8% [61]. The multivariate regression analysis demonstrated that levels of LDL-cholesterol (OR = 4.380, 95% CI = 1.101–17.419, *p* = 0.036) and miR-455-5p (OR = 0.154, 95% CI = 0.039–0.606, *p* = 0.007) were independently associated with the severity of ICAS. Moreover, miR-455 (HR = 0.128, 95% CI = 0.029–0.573, *p* = 0.007), likewise the degree of ICAS, occurred important risk factors for ICAS-related IS [61]. The Kaplan–Meier survival curves showed that low miR-455-5p expression was related to the incidence of IS during follow-up (log Rank P = 0.002).

In the study by Dolz et al., the authors focused on the progression of asymptomatic ICAS and the potential role of miRNAs as non-invasive biomarkers of carotid plaque instability and ICAS progression [121]. The analysis identified a different miRNA expression profile in RNA samples from patients with ICAS progression during the follow-up period, compared to the patients without ICAS progression. Progression of ICAS was associated with significantly higher expression levels of miR-199b-3p (0.056; IQR, 0.014–0.958 vs. 0.029; IQR, 0.002–1.139; *p* = 0.049), miR-130a-3p (0.022; IQR, 0.007–0.177 vs. 0.006; IQR, 0.001–0.197; *p* = 0.042) and miR-24-3p (0.126; IQR 0.025–1.978 vs. 0.053; IQR, 0.007–0.95; *p* = 0.028) [121]. However, in the multivariate analysis, only the overexpression of a proangiogenic miR-130a-3p was independently associated with the ICAS progression (ORs, 5.4; 95% CI, 1.03–28.2; *p* = 0.025) and IS risk [121].

In line with this, miR-637 was assessed as a potential biomarker of ICAS and IS occurrence [122]. The prospective analysis revealed that down-regulation of miR-637 was associated with the degree of ICAS, with a sensitivity of 85.6%, and a specificity of 83.3% for ICAS of at least 50% lumen narrowing [122]. During the 5 year follow-up, the Kaplan–Meier analysis showed that the lower miR-637 levels were associated with a higher incidence of ICAS-related IS (HR = 0.073, 95% CI = 0.017–0.313, *p* < 0.001), along with severe ICAS (HR = 0.144, 95% CI = 0.045–0.463, *p* = 0.001) [122]. The study proved that the miR-637 downregulation is responsible for cardiovascular events in patients with atherosclerosis [137] by promoting the proliferation and migration of VSMCs by regulating IGF-2 [138].

In another study by Zhu et al., the sensitivity and the specificity of miR-486 for a diagnosis of significant asymptomatic ICAS were 82.4% and 89.7%, respectively [123]. The results showed that HAEC apoptosis was weakened by increased miR-486-5p, reflecting its protective effect against vascular ECs injury [123]. Vascular ECs injury may promote the release of intracellular adhesion factors and then trigger the transformation of macrophages into foam cells. In vitro experimental results demonstrated that miR-486-5p can also inhibit the release of ICAM-1 in HAECs. Besides its regulatory role in the apoptosis of vascular ECs, the findings of the study also demonstrated the influence of miR-486-5p on inflammation and oxidative stress [139].

There is limited data on the miRNAs acting as triggers for symptom development in patients with a significant ICAS. In the study by Grosse et al., the levels of several popular circulating miRNAs (miR-92a, miR-126, miR-143, miR-145, miR-155, miR-210, miR-221, miR-222, and miR-342-3p) were analyzed in patients with symptomatic and asymptomatic ICAS that were referred to CEA [124]. The study has not found miRNAs diagnostic for symptomatic ICAS, except from the trend to difference for miR-92a in multivariate analysis [124]. The authors have not observed either the relationship between miRNAs expression levels and micro embolic signals that correspond to plaque fragility and distal embolization to cerebral arteries by debris released from the plaque [140].

The study by Luque et al. aimed to present the associations between atherosclerotic plaque instability and circulating miR-638 level [125]. The results showed significantly lower miR-638 levels in patients with symptomatic ICAS, as compared to asymptomatic ICAS [125]. The study demonstrated that miR-638 level was correlated with the IS incidence and IS recurrence, smoking habit, bilateral ICAS, coronary artery disease, and hypercholesterolemia [125]. The authors considered miR-638 as a potential marker associated with plaque vulnerability and IS.

In the study by Badacz et al., the differences in expression levels of circulating miR-133a, miR-124, and miR-134 were found in patients with symptomatic vs. asymptomatic ICAS [83]. The authors observed higher serum levels of miR-1 (*p* = 0.032) in a thrombotic plaque, as well as higher levels of miR-1 (*p* = 0.04) and miR-16-5p (*p* = 0.003) in patients with ulcerated plaques [83].

Similarly, in the study by Zhang et al., the expression levels of miR-106b-5p were analyzed in asymptomatic ICAS patients [126]. The expression of miR-106b-5p was associated with hypertension, dyslipidemia, and degree of ICAS. In ROC analysis, miR-106b-5p showed a good predictive value for significant ICAS compared to healthy individuals, with a sensitivity of 89.7% and a specificity of 83.6% [126]. Furthermore, patients with higher miR-106b-5p expression levels were more likely to suffer from IS during follow-up (log rank *p* = 0.020; HR 5.431, 95% CI: 1.592–18.520, *p* = 0.007).

The other study by Zhang et al. focused on miR-637 and its possible role as a biomarker for IS risk [122]. The multivariate analysis revealed that downregulation of miR-637 independently predicted future IS (HR = 0.073, 95%CI = 0.017–0.313, *p* < 0.001), thus making the analyzed miRs possible biomarkers in ICAS patients [122].

In line with this, Li et al. analyzed expression levels of miR-483-5p in patients with asymptomatic ICAS and evaluated its diagnostic and predictive value for IS [127]. The results revealed that patients with high miR-483-5p levels had higher a risk of IS (log-rank *p* = 0.011), as compared to patients with lower miR-483-5p expression levels (HR 2.670; 95% CI 1.099–6.484; *p* = 0.030) [127]. Similarly, miR-342-5p was proposed as an IS risk predictor at a 5 year follow-up by Zhou et al. [128].

Several more studies found possible predictive value of specific miRNAs for the onset of IS. Chen at al. stated that elevated miR-92a expression could differentiate patients with asymptomatic ICAS from healthy subjects, and was an independent predictive factor for the occurrence of IS (HR = 2.971, 95% CI = 1.230–7.173, *p* = 0.015) [129], while Zhou et al. observed miR-342-5p as a potential predictor of IS in asymptomatic patients with ICAS (HR = 5.512, 95% CI = 1.370–22.176, *p* = 0.016) [129].

Similar results were observed for miR-19a-3p by Liu et al. who stated that higher expression of serum miR-19a-3p (HR = 8.507, 95% CI = 2.239–32.328, *p* = 0.002) and degree of ICAS (HR = 3.695, 95% CI = 1.127–12.109, *p* = 0.031) were independent predictors for the onset of IS [130], whereas Lu et al. found miR-27b an important risk factor of IS (HR = 5.067, 95% CI = 1.170–21.943, *p* = 0 .030) [131].

Conversely, Li et al. observed that a downregulation of miR-206 was an independent risk factor for the development of IS during a 5 year follow-up (HR = 0.046, 95% CI = 0.005–0.431, *p* = 0.007) [132]. In the study by Liu et al., low miR-9-5p expression levels (HR = 0.239; 95% CI = 0.087–0.652, *p* = 0.005) were associated with IS [133].

Finally, the recent study by Badacz et al. analyzed cardiovascular outcomes in patients with diabetes who underwent intervention on ICAS [134]. During a follow-up period of 7 years, miR-134-5p occurred an independent risk factor of CVD/MI/recurrent IS (HR = 1.12; 95% CI = 1.05–1.21, *p* < 0.001) in diabetic patients, while miR-16 in patients without diabetes [134]. The authors suggested the diversity of miRNAs profile in patients with and without diabetes for cardiovascular outcomes. In line with this, Sardu et al. found miR-24 as a possible predictor of ischemic cardiovascular events and hospitalization for heart failure in pre-diabetic patients at a 2 year follow-up [141].

In addition to miRNA, there is probably a huge role for microparticles in vascular damage in diabetic patients [142]. Some of these macromolecules have angiogenetic properties or participate in modulation of vascular senescence or remodeling, taking part in the process of vascular aging [142]. The other miRNAs that could be potentially associated with plaque instability and cerebrovascular outcomes are: miR-21, miR-221, miR-133b, and miR-124 were independent predictors (Table 2) [135,136].

## 4. microRNA Expression Levels in Plaques Removed during CEA

Apart from circulating serum or plasma miRNAs, the carotid plaque can contain large amounts of miRNAs. These miRNAs often differ or have diverse expression levels in plaque, compared to systemic miRNAs [97]. Intra-plaque miRNAs play the role in the process of carotid plaque evolution and progression, leading to plaque rupture and IS, as well [97]. These miRNAs can be explored only when a biological material is available, e.g., after the excision of carotid plaque that takes place during CEA.

The study by Cipollone et al. covered the large-scale miRNA analysis of symptomatic and asymptomatic carotid plaques excised during CEA (Table 3) [97]. The results presented significant up-regulation of several miRNAs (out of 41 analyzed), miR-145 and miR-133a in particular, in symptomatic plaques as compared to asymptomatic ones [97].

In the study by Maitrias et al., seven miRNAs were dysregulated, including miR-100, miR-125a, miR-127, miR-133a, miR-145, and miR-221 in carotid plaques obtained during CEA in 30 symptomatic and asymptomatic patients [22]. The identified miRNA profile was corresponding to the inflammatory process leading to plaque destabilization. miR-100, miR-125a, and miR-127 were proven to modulate the vascular inflammation and angiogenesis, as well as oxidative stress [125,146,147,148]. Finally, miR-133a, and miR-145 are considered to play the key role in regulating the vascular remodeling and inflammation especially by controlling VSMCs proliferation and differentiation [149,150]. In the study by Bazan et al., the miR-221 and miR-222 expressions were evaluated in carotid plaques obtained from CEA in three groups of patients: with asymptomatic ICAS, symptomatic ICAS (neurologic symptoms within 5 days), and patients who underwent neurologic event earlier [143]. Patients in acute phases of neurologic symptoms related to ICAS exhibited a significant decrease in miR-221 and miR-222 expression as compared to the other groups [143,150]. This confirms the direct association of miRNAs dysregulation in the mechanisms of plaque instability [143].

Another promising potential biomarker of plaque instability in carotid atherosclerosis is miR-200c [144]. The miR-200c plaque concentration was up-regulated in carotid plaques that presented unstable features on imaging studies. Additionally, miR-200c positively correlated with instability biomarkers, such as COX2, IL-6 or MMP-9 [144]. miR-146a and pro-inflammatory cytokines in patients with severe ICAS were addressed in the study by Huang et al. [151]. The expression levels of miR-146a, IL-6, and TNF-α in the ICAS group were higher than those in the control group and positively correlated with the degree of ICAS and plaque vulnerability [151]. In the study by Wei et al., upregulation of miR-330-5p level was identified in symptomatic carotid plaques [145].

Thus, the complex process of plaque destabilization is attributed to both intra-plaque action of miRNAs and is simultaneously regulated by various circulating miRNAs.

## 5. Conclusions

MicroRNA have become a point of interest in recent years of scientific research including broad range of pathophysiological issues. Multiple recent studies proved miRNAs to be the important factor of atherosclerotic process, including coronary, peripheral, or carotid artery disease. miRNA seem to be directly associated with a patient’s atherosclerosis severity and functional prognosis, suggesting that miRNAs can be used as potential biomarkers for the diagnosis and prognosis evaluation of cardiovascular events. According to modern evidence, some miRNAs reveal a potential diagnostic or prognostic value in revealing the potential vulnerable plaque features or predicting future ischemic events.

Several miRNAs were proven to be a viable diagnostic tool in assessing the risk of carotid plaque destabilization, and as a result of plaque rupture, the risk of IS occurrence is high. Moreover, the studies have shown that different miRNAs were able to differentiate the symptomatic and asymptomatic course of ICAS.

However, the studies struggle with a wide range of miRNAs, missing identification of the narrow group of pivotal and key miRNAs responsible for the atherosclerosis processes, which may play the role of biomarkers in everyday routine practice. Therefore, dedicated study is necessary in order to establish which miRNAs could be used in carotid atherosclerosis.

## Figures and Tables

**Table 1:** Critical miRNAs participating in atherosclerotic carotid artery lesions development: from fatty streaks to mature plaque: a therapeutic approach.

Critical Stages in Atherosclerosis	miRNA	Mechanism	Effect of miRNA Action	Therapeutic Approach (HUVEC or Animal Studies)	Ref.
Initiation and early atherosclerosis					
‘Brakes’ of atherosclerosis					
Promotes ECs proliferation and repair, protects ECs	miR-126-5p	suppression of the Notch1 inhibitor Dlk1	At non-predilection sites, high miR-126-5p levels in ECs confer a proliferative reserve that compensates for the antiproliferative effects of hyperlipidemia	T, injection of miR-126-5p rescued ECs proliferation at predilection sites and limited atherosclerosis	[29]
Decreases atherosclerosis progression	miR-155	downregulation of MAP3K10 downregulation of ET-1 and ANG II type I receptor	Down-modulates inflammatory cytokine production	T, the miR-155 mimic decreased IL-6, MMP-9 and TNF-α secretions of oxLDL-induced macrophages	[31,32]
Decreases lipid uptake in macrophages, inhibits endothelial activation	miR-146a	regulates TLR4, increases eNOS expression	Inhibits ox-LDL and inflammatory response (decreases IL-6, -8, MMP-9)	Overexpression may be useful	[35]
Macrophage polarization	MiR-125a	downregulation of sVEGFR1	Decreases lipid uptake in macrophages, modulates extracellular VEGF by manipulating sVEGFR1	T, miR-125a-5p inhibition reduces VEGF through the increased sVEGFR1	[36]
Increase reverse cholesterol transport from macrophages to the liver for excretion	miR-206 miR-223	promote efflux promote efflux	crucial for the prevention of lipid accumulation and atherosclerosis	T, these miRs can be efficiently delivered to macrophages via chitosan nanoparticles	[39,40]
Prevents ECs senescence	miR-let-7g	Stimulates anti-aging gene SIRT1, and IGF 1, inhibits expression of LOX-1	exert anti-aging effects on ECs	T, antagonizing endogenous let-7 has induced cell proliferation	[42]
Prevents ECs senescence	miR-143	targets a network of transcription factors, including KLF4, myocardin, and Elk-1	promotes differentiation and repress proliferation of VSMCs	microvesicles containing miR-143 injected into mice could reduce the formation of atherosclerotic plaques	[43,44]
Suppresses atherosclerotic plaque formation	miR-520	targets RelA/p65	regulates VSMCs decreasing migration and proliferation	miR-520c-3p agomir decreased atherosclerotic plaque size	[45]
High expression is needed to maintain a contractile phenotype of VSMCs	miR-22	multiple target genes	induce the phenotypic switch from synthetic to contractile	T, the stent with the miR-22 coating showed significant capability to inhibit in-stent restenosis	[46]
Promotors of atherosclerosis					
Increases endothelial inflammation	miR-92a	regulation of KLF2	markedly enhanced by hypercholesterolemia	T, inhibition of miR-92a reduces endothelial inflammation and atheroma plaque size	[47]
Vascular senescence, vascular calcifications Altered lipid metabolism Increases inflammatory cytokines secretion of macrophages M1	miR-34a	inhibition of SIRT1 and AXL receptor tyrosine kinase targets cholesterol transporters: ABCA1 and ABCG1 through the nuclear hormone LXRα	aggravates and accelerates vascular senescence increase the binding capacity of oxLDL to macrophages stimulate pro-inflammatory cytokines (TNF-α, IL-1β, IL-6, IL-12, IL-23), and chemokines (CCL5, CCL8, CXCL2, CXCL4)	T, inhibition with antago-miR-34a	[48,49]
Promotes cholesterol accumulation in macrophages, decreases reverse cholesterol transport	miR-33a	Targets hepatic ABCA1	inhibit efflux, increases macrophages ox-LDL uptake, foam cells accumulation	T, inhibition of miR-33a facilitates atherosclerosis regression	[50,51]
Promotes atherosclerosis	miR-155	repressing Bcl6 in macrophages, suppress eNOS	increases pro-inflammatory NF-κB signaling, down-regulates the expression of eNOS and production of NO	T, inhibition of miR-155 increased eNOS expression and NO production	[32,52]
Increases apoptosis in ECs	miR-17-5p	repression of ABCA1 expression through directly binding to its 3′-UTR	rate of apoptosis in ECs	T, inhibition of miR-17 suppresses apoptosis, hence decrease infarct size area, and improves microcirculation of the heart tissue, decreasing heart failure symptoms	[53,54]
Promotion of monocyte adhesion, proinflammatory Lipid metabolism	miR-21	targets PPAR α targets TLR4 and NF-κB	enhances the expression of VCAM-1 and MCP-1 and the adhesion of monocytes to ECs LPS-induced lipid accumulation and inflammatory response in macrophages	Overexpression of miR-21 up-regulated AP-1 activation, which was attenuated by exogenous expression of PPARα overexpression of miR-21 significantly decreased the secretion of IL-6 and increased IL-10 levels	[55,56]
Induces ECs apoptosis, development of atherosclerosis	miR-142-3p	up-regulation of Rictor and the Akt/eNOS	atherosclerosis-associated ECs apoptosis	T, the antagomir-142-3p attenuated endothelial apoptosis and retarded the atherosclerosis progression in the aorta of ApoE-/- mice	[57]
Increase pro-inflammatory cytokines	miR-342-5p	targets Akt1	induces proinflammatory mediators such as NOS2 and II6 in macrophages via the upregulation of miR-155	T, the miR-342-5p antagomir upregulated Akt1 expression and suppressed the expression of miR-155 and Nos2	[58]
Mature plaques					
Marker of response to clopidogrel, targets P2Y12 receptor	miR-223-3p	possible P2Y12 site targeting	on-clopidogrel platelet reactivity	decreased miR-223 expression was a predictor of low responders to clopidogrel	[41]
Plaque stabilization	miR-145	targets KLF4,5	VSMCs contractility, increase fibrous cap area, reduce the necrotic core area	T, delivery of miR-145 may limit atherosclerotic plaque growth, and restore contractile levels in VSMCs	[59,60]
Macrophage polarization	miR-455	targets SOCS3	decreased expression leads to ECs injury induced by ox-LDL	T, overexpression with miR-455 inhibits apoptosis, migration of VSMCs, and lowers ox-LDL	[61]
Marker of platelet activation, targets COX-1 receptor through the regulation of TXS	miR-34b-3p	targets TBXAS1	miR-34b-3p may regulate the platelet response by suppressing TBXAS1 expression and megakaryocyte proliferation	T, miR-34b-3p may facilitate the antiplatelet efficiency of aspirin through inhibiting TBXAS1	[62]
Responsive to antiplatelet therapy	miR-126-3p	affects ADAM9 and P2Y12 receptor expression	Increases platelets aggregation	T, antagomiR against miR-126-3p reduces platelets aggregation	[63]
Decreases size of atherosclerotic lesions, alleviate ox-LDL-induced ECs injury, angiogenesis and vascular integrity	miR-126-3p	activation of VEGF and NF-kB signaling	decreased expression in advanced carotid plaques with high discriminating value (AUC: 0.998)	patients with severe carotid stenosis demonstrated down-regulation of miR-126	[64]
Plaque stabilization	miR-210	targets the APC gene, affecting Wnt signaling and regulating VSMCs survival	enhances fibrous plaque stability in mature plaques	T, miR-210 mimics prevent carotid plaque rupture; modulating miR-210 improved fibrous cap stability	[65]
Promotes atherosclerosis growth	miR-103-3p	targets KLF4	stimulates inflammatory activation, and uptake of oxidized LDL cholesterol	T, reduction in miR-103 levels results in the reduction of atherosclerosis and endothelial inflammation	[66]
Decreases ECs regeneration and repair	miR-652-3p	suppression of the endothelial repair gene Ccnd2	inhibits ECs regeneration and repair following mechanical injury	downregulates Ccnd2 in endothelial cells, lowering cell proliferation	[67]
Plaque stabilization	miR-223	targets TLR4	reduces foam cell formation, and production of pro-inflammatory cytokines	Overexpression decreases lipids deposition and inflammation	[68]
Plaque instability	miR-92a-3p	SIRT1, H_2_O_2_-induced changes in VSMCs	increased apoptosis, oxidative stress, CIMT, and pro-inflammatory MMP-9,	miR-92a overexpression regulates the expression levels of MMP-9 and TIMP3	[69,70]
Plaque instability	miR-133a	Matrix metallopeptidase 9	inhibits the proliferation of VSMCs and induces apoptosis	the miR-133a-3p mimic inhibited proliferation and promoted VSMC cell apoptosis	[71]
Promotes endothelial migration	miR-486	targets HAT1	induces apoptosis and oxidative stress, pro-atherosclerotic, affects endothelial migratory activity	Inhibition of miR-486 limits foam cell formation by increasing cholesterol efflux	[69,72]
Increases pro-inflammatory cytokines	miR-331	down-regulation of SOCS1	a pro-inflammatory response in atherosclerotic plaques	miR-331 suppression causes up-regulation of SOCS1 and anti-inflammatory mechanism in atherosclerosis	[73,74]
Plaque stabilization	miR-100	down-regulation of E-selectin and VCAM-1	miR-100 restrains vascular inflammation in vitro and in vivo by suppressing endothelial adhesion molecule expression and thereby attenuating leukocyte–endothelial interaction	Inhibition of miR-100 Stimulates Atherogenesis in Mice	[75]
Plaque instability	miR-105	transported via HDL	overexpression of miR-105 in patients with familial hypercholesterolemia	HDL can deliver miRNA-105 to recipient cells, contributing to altered gene expression	[76]
Plaque instability	miR-155	Targets the transcription factor HBP1	Increase macrophages, foam cells content, and necrotic core in plaques	T, inhibition of miR-155 reduced necrotic core, apoptosis, and prevented progression of atherosclerosis	[77,78]
Plaque instability	miR-124	Targets P4HA1	Inhibits collagen synthesis in atherosclerotic plaques	Overexpression of miR-124 increased the expression of anti-inflammatory cytokines by binding p38 signaling pathway	[79,80]
Plaque instability	miR-134	ANGTPL4/LPL	associated with chronic inflammation (hs-CRP and TNF-α), cholesterol mass, and plaque vulnerability features on ultrasonography	T, lower LPL activity with inhibitors of miR-134	[81,82,83]
Lipometabolism	miR-122	inhibits AMPK and SIRT1	correlated with TC, TG, and LDL-C levels	serum level of miR-122 correlated with atherosclerotic severity	[84]

ABCA1: ATP-binding cassette subfamily A member 1; ABCG1: ATP-binding cassette subfamily G member 1; Akt: protein kinase B; AMPK: Adenosine monophosphate–activated protein kinase; ANG II: angiotensin II; ANGTPL/LPL: Angiopoietin/lipoprotein lipase; CIMT: carotid intima-media thickness; Dlk1: delta-like 1 homolog; eNOS: nitric oxide synthase; ECs: endothelial cells; ET-1: Endothelin 1; H2O2: hydrogen peroxide; HAT1: Histone acetyl-transferase 1; HMG-box transcription factor 1; hs-CRP: high-sensitivity C-Reactive Protein; IGF: insulin growth factor; KLF: Kruppel-like factor; LXRα: Liver X receptor α; LPS: lipopolysaccharide; MAP3K10: mitogen-activated protein 3 kinase 10; MCP-1; monocyte chemotactic protein-1; MMP: metalloproteinase protein; n/d: no data available; NF-κB: nuclear factor-κB; P4HA1: prolyl 4-hydroxylase subunit alpha-1; PPARα: peroxisome proliferators-activated receptor-α, RelA/p65: REL-associated protein involved in NF-κB heterodimer formation p65 subunit; sVEGFR1: soluble VEGF receptor-1; SIRT1: sirtuin 1; SOCS1: suppressor of cytokine signaling 1; T: therapeutic approach; TBXAS1: thromboxane synthase thromboxane A synthase 1; TLR4: toll-like receptor 4; TNF-α: tumor necrosis factor alpha; VCAM-1: vascular cell adhesion molecule-1; VSMC: vascular smooth muscle cells; VEGF: vascular endothelial growth factor.

**Table 2:** **Serum/plasma** microRNAs that are potentially diagnostic for the degree of ICAS, can differentiate between patients with asymptomatic versus symptomatic ICAS, or are prognostic for risk of ICAS-related IS.

Study Participants	microRNA	Down- vs. Up-Regulated	Rationale for the Use of the miRNA	Statistical Analysis AUC, or HR (95% CI), *p*-Value	Ref.
30 patients with aICAS (≥50%) 20 patients with non-obstructive carotid plaques	miR-29a-3p miR-33a-3p miR-126-3p miR-126-5p	Down Up Down Down	Regulates VSMCs function, inflammation Cholesterol metabolism ECs repair and proliferation ECs repair and proliferation	For 50% ICAS or greater: AUC 0.79, *p* = 0.035 AUC 0.68, *p* < 0.001 AUC 0.78, *p* < 0.001 AUC 0.89, *p* < 0.001	[100]
70 patients with aICAS (≥50%) 65 patients with non-obstructive carotid plaques	miR-455	Down	Neuroprotective, anti-inflammatory, inhibits apoptosis and the migration of VSMCs, lowers ox-LDL	For 50% ICAS or greater: AUC 0.927, *p* < 0.001 Risk factor for IS: 0.128 (0.029–0.573), *p* = 0.007	[61]
67 patients with sICAS (≥50%) 27 patients with aICAS (≥50%)	miR-124 miR-133a miR-134	Up Down Up	associated with inhibited proliferation of VSMCs and colagen syntesis, increased apoptosis	The expression levels of miR-124 (*p* = 0.036) and miR-134 (*p* = 0.02) were upregulated in sICAS, whereas miR-133a level was down-regulated (*p* = 0.043)	[83]
65 patients with sICAS (≥50%) 27 patients with aICAS (≥50%)	miR-133b	Up	the incidence of IS at 3 y.	risk of IS: 2.25 (1.01–5.02), *p* = 0.047	[83]
60 patients with aICAS (≥50%)	miR-130a-3p	Up	a proangiogenic miRNA	For ICAS progression: 5.4 (1.03–28.2); *p* = 0.025	[121]
97 patients with aICAS (≥50%) 90 healthy individuals	miR-637	Down	vascular-related diseases such as atherosclerosis, ischemic stroke, and hypertension	For 50% ICAS or greater: 0.050 (0.014–0.174), *p* < 0.001	[122]
97 patients with aICAS (≥50%) 90 healthy controls	miR-637	Down	the incidence of IS at 5 y.	risk of IS: 0.073 (0.017–0.313), *p* < 0.001	[122]
91 patients with aICAS (≥50%)	miR-486-5p	Down	association with the cardiovascular risk score; prediction of ICAS progression	For 50% ICAS or greater: AUC 0.921, *p* < 0.001	[123]
21 patients with sICAS (≥50%) 23 patients with aICAS (≥50%)	miR-92a miR-126 miR-143 miR-145 miR-155 miR-210 miR-221 miR-222 miR-342-3p	Up NS NS NS NS NS NS NS NS	miRs potentially associated with atherosclerosis	The level of miR-92a was higher in sICAS compared to aICAS in T-student test (*p* = 0.046), but failed in multivariate analysis no statistical difference for the other miRs	[124]
22 patients with sICAS (≥50%) 36 patients with aICAS	miR-638	Down	miR expressed in VSMCs, proliferative vascular diseases, the incidence of IS at 5 y.	Risk of IS: AUC 0.66, *p* = 0.04	[125]
58 patients with aICAS (≥50%) 61 healthy controls	miR-106b-5p	Up	miR as independent factor for the high degree of ICAS	For 50% ICAS or greater: 6.582 (1.549–27.963), *p* = 0.011	[126]
58 patients with aICAS (≥50%) 61 healthy controls	miR-106b-5p	Up	role in the regulatory network of atherosclerotic gene expression, the incidence of IS at 5 y.	Risk of IS: 5.431 (1.592–18.520), *p* = 0.007	[126]
128 patients with aICAS (≥50%) 76 healthy controls	miR-483-5p	Up	inhibitory effect on angiogenesis the incidence of IS at 5 y.	risk of IS: 2.670 (1.099–6.484), *p* = 0.030	[127]
92 patients with aICAS (≥50%) 86 healthy controls	miR-342-5p	Up	the incidence of IS at 5 y.	risk of IS: 5.512 (1.370–22.176), *p* = 0.016	[128]
122 patients with aICAS (≥50%) 67 healthy controls	miR-92a	Up	plaque instability the incidence of IS at 5 y.	risk of IS: 2.971 (1.230–7.173), *p* = 0.015	[129]
101 patients with aICAS (≥50%) 98 healthy controls	miR-19a-3p	Up	inflammation and apoptosis the incidence of IS at 5 y.	risk of IS: 8.507 (2.239–32.328), *p* = 0.002	[130]
71 patients with aICAS (≥50%) 58 healthy controls	miR-27b	Up	higher expression in atherosclerosis the incidence of IS at 5 y.	risk of IS: 5.067 (1.170–21.943), *p* = 0.030	[131]
105 patients with aICAS (≥50%) 101 healthy controls	miR-206	Down	miR correlation with ICAS degree the incidence of IS at 5 y.	For 50% ICAS or greater: 0.336 (0.132–0.857), *p* = 0.022 risk of IS: 0.046 (0.005–0.431), *p* = 0.007	[132]
88 patients with aICAS (≥50%) 86 healthy controls	miR-9-5p	Down	development of atherosclerosis the incidence of IS at 5 y.	risk of IS: 0.239 (0.087–0.652), *p* = 0.005	[133]
37 diabetic patients with sICAS (≥50%) 64 non-diabetic patients with sICAS (≥50%)	miR-134 miR-16	Up Up	CVD/MI/re-IS in diabetic vs. non-diabetic patients at 7 y.	miR-134 associated with risk in DM: 1.12 (1.05–1.21), *p* < 0.001 miR-16 associated with risk in non-DM: 1.01 (1.001–1.012), *p* = 0.016	[134]
167 patients with sICAS (≥50%) 66 patients with aICAS (≥50%) 157 healthy controls	miR-21 miR-221	Up Down	plaque instability predictors the incidence of IS at 5 y.	risk of IS: miR-21: HR: 6.2, *p* < 0.001 miR-221: HR: 10.4, *p* < 0.001	[135]
73 patients with features of stable plaque on US 87 vulnerable plaques on US	miR-124	Up	plaque instability predictor	Associated with vulnerability AUC 0.853, *p* < 0.001	[136]

aICAS: asymptomatic internal carotid artery stenosis; AUC: area under the curve; CI: confidence interval; CVD/MI/re-IS: cardiovascular death/myocardial infarction/recurrent ischemic stroke; HR: hazard ratio; NS: non-significant; OR: odds ratio; sICAS: symptomatic internal carotid artery stenosis; US: ultrasonography; y.: years.

**Table 3:** Intra-plaque microRNAs associated with carotid plaque vulnerability and rupture.

Study Groups	microRNA	Down- vs. Up-Regulated	Rationale for Use of Individual microRNA	Statistical Analysis	Reference
15 plaques from sICAS 15 plaques from aICAS	miR-100, miR-125a, miR-127, miR-133a, miR-145, miR-221	Up Up Up Up Up Up	microRNAs involved in plaque growth and instability	Difference between sICAS vs. aICAS: *p* < 0.001 *p* < 0.001 *p* < 0.001 *p* < 0.001 *p* < 0.001 *p* < 0.001	[22]
22 plaques from sICAS 31 plaques from aICAS	miR-145 miR-133a	Up	large-scale analysis	Difference between sICAS vs. aICAS: *p* = 0.027 *p* = 0.044	[97]
45 plaques from sICAS 31 plaques from aICAS	miR-221 miR-222	Down Down	miRs promoting VSMC proliferation and intimal thickening	Time to neurological symptoms onset: R2 = 0.44, *p* < 0.001 R2 = 0.45, *p* < 0.001	[143]
12 plaques from sICAS 10 plaques from aICAS	miR-200c	Up	induction of endothelial dysfunction, ROS production	Difference between sICAS vs. aICAS: *p* < 0.001	[144]
20 plaques from sICAS Qualified as stable vs. unstable in histological assessment	miR-330-5p	Up	regulatory effect of miRNA-330-5p on Talin-1 mediator	Difference between sICAS vs. aICAS: *p* < 0.05	[145]

aICAS: asymptomatic carotid artery stenosis; sICAS: symptomatic carotid artery stenosis; ROS: reactive oxygen species; CEA: carotid endarterectomy; VSMC: vascular smooth muscle cells; TNF-α: tumor necrosis factor alpha; IL-1β: interleukin 1-beta; AUC: area under the curve; CI: confidence interval.

## Data Availability

The data presented in this study are available on request from the corresponding author. The data are not publicly available due to privacy.
